# Community-based HIV prevention services for transgender people in Ukraine: current situation and potential for improvement

**DOI:** 10.1186/s12913-023-09656-5

**Published:** 2023-06-14

**Authors:** Oleksandr Neduzhko, Tetiana Saliuk, Oksana Kovtun, Nadiia Semchuk, Olga Varetska

**Affiliations:** 1 Ukrainian Institute on Public Health Policy, 5 Biloruska Str. Office 20, 27, Kyiv, 04050 Ukraine; 2 Alliance for Public Health, Kyiv, Ukraine

**Keywords:** Transgender people, HIV prevention, Community-based services, Gender-affirming care

## Abstract

**Background:**

Community-based HIV-prevention services are a key approach to prevent HIV transmission among key population representatives. Transgender people have multiple specific needs and it is crucial to use prevention approaches that effectively respond to those needs and facilitate barriers on the way to use HIV prevention and related services. This study is aimed to explore the current state of community-based HIV prevention services among transgender people in Ukraine, its limitations and potential for improvement based on the experience and perceptions of transgender people, physicians, and community social workers providing services to transgender people.

**Methods:**

Semi-structured in-depth interviews were conducted among physicians providing services to transgender people (N = 10), community social workers (N = 6), and transgender people (N = 30). The objectives of the interviews were to explore: the relevance of the community-based HIV prevention services to the needs of transgender people; the key components of the most preferred *(“ideal”)* HIV-prevention package for transgender people; ways to optimize the existing HIV prevention package for transgender people including enrollment and retention. Systematically collected data were analyzed and coded into the main domains, thematic categories and subcategories using thematic analysis.

**Results:**

The current HIV prevention programs were well-evaluated by the majority of respondents. Gender-affirming care was found to be the key need of transgender people. Integration of HIV prevention services and gender-affirming care was perceived as the main way to address the needs of transgender people. Internet-based and peer word-of-mouth recruitment may improve enrollment in services. Optimization of existing HIV prevention package may include: psychological counseling, referral and navigation to medical services, legal services, pre- and post-exposure prevention, dissemination of tube lubricants, femidoms and latex wipes, use of oral fluid test systems for HIV self-testing.

**Conclusions:**

The findings of this study suggest potential solutions to improve community-based HIV prevention services for transgender people by introducing a transgender people -oriented package, which integrates gender transition, HIV prevention and other services. Provision of prevention services based on assessed risk and referral/navigation to related services are the key options for optimization of the existing HIV prevention package.

**Trial registration:**

Not applicable.

## Background

The gender identity of transgender people (TP) is different from the sex assigned at birth [1]. The terms transgender man (TM), and transgender woman (TW) will be used in this article to refer to gender identity. TP experience psychological distress called gender dysphoria which is caused by a discrepancy between a person’s gender identity and sex assigned at birth [1].

Gender affirmation could be used to address gender dysphoria [2, 3] and consists of three main dimensions: (1) social (name pronoun); (2) medical (hormones and cross-sex surgeries); and (3) legal (change of the name) [4]. Gender affirmation can be fulfilled through gender transition process and may include combination of social, medical and legal changes [4]. According to the Ukrainian Unified Clinical Protocol of *“Primary, Secondary and Tertiary Medical Care: Gender dysphoria”* [5], a diagnosis of gender identity disorder [6] requires outpatient follow-up for two years or a two-week inpatient examination. The diagnosis of gender identity disorder allows TP to receive hormone therapy and surgery. After a medical advisory commission meeting with the participation of a family doctor and other specialists, *“Medical certificate of gender reassignment”* may be issued. This certificate allows TP to apply to civil registry offices to replace the birth certificate, so that gender at birth corresponds to gender self-identification. Complex plastic surgeries, such as phalloplasty and vaginoplasty are not performed in Ukraine. Primary medical care and HIV care is free in Ukraine, the National Health Service of Ukraine pays for the provision of the guaranteed package [7, 8].

The estimated worldwide HIV prevalence among TW was 19.1% [9] and 6.8% among TM [10]. The results of the review suggest a new wave of HIV epidemic in Ukraine, not linked to injection practices [11]. The results of the biobehavioral study (IBBS) conducted in Ukraine among TP in 2020 demonstrate 2% HIV prevalence among TW [12]. The estimated number of TP in Ukraine is 8,200, estimations on specific gender identities are not available [13].

HIV prevention for TP is inadequate [9], little is known on how to address the needs of TP through non-governmental organizations (NGOs) [14]. Global Fund is the main source of funding for HIV prevention programs in Ukraine, which are implemented by the International Charitable Foundation “Alliance for Public Health” and through the network of local NGOs [15]. In 2020, these programs reached 2,734 TP in Ukraine. Based on the results of operational research conducted in 2014, the HIV prevention package previously developed to address the needs of three key populations (KPs: people who inject drugs, commercial sex workers, and men who have sex with men) was adapted to meet the needs of TP [16]. It includes condoms and lubricants distribution, counseling of social workers and health professionals, testing for HIV, syphilis, hepatitis C, and early tuberculosis detection using a paper-based questionnaire.

This study aimed to explore the relevance of community-based HIV prevention services in Ukraine to the needs of TP; key components of the most preferred *(“ideal”)* HIV-prevention package; and ways to optimize the existing HIV prevention package for TP. The results of this study can be used to advocate and promote TP HIV prevention and care needs.

## Methods

### Study overview

This qualitative study was conducted between January 9 - March 18, 2020, in two Ukrainian cities - Odesa and Kyiv. In-depth interviews were conducted with physicians (n = 10), community social workers (n = 6) and TP (n = 30).

### Recruitment

Purposeful sampling was used to recruit respondents. Physicians interested in participating in the survey were identified in cooperation with two local NGOs providing services for TP – “Partner” NGO (Odesa) and – “Convictus Ukraine” NGO (Kyiv). Social workers were recruited from the mentioned NGOs.

TP were recruited from those representatives who previously participated in an IBBS among TP in Ukraine. The recruitment of the participants was based on several inclusion criteria: assigned TW or TM, 14 years of age or older, residence in one of the study cities, previous IBBS participant status, and provided informed consent. TM were recruited in Kyiv and TW were recruited both in Kyiv and Odesa. Given possible differences in the perceptions and experience of using HIV prevention services among different TP subgroups, a targeted stratified sampling approach was implemented by gender, HIV prevention services experience, and city of residence to address major variations [17].

### Data collection

Semi-structured one-to-one in-depth interviews were conducted using interview guides that were developed in consultations with the physicians, social workers and TP. Interview guides for TP and social workers included: general questions on HIV prevention services; NGO-based HIV prevention services; enrollment in NGO-based HIV prevention services and related services. Interview guide for physicians was mostly focused on medical services including general care, transgender transition services, protection from violence and assistance to victims of violence.

All respondents provided verbal informed consent and completed a brief demographic questionnaire afterwards. Each participant who completed the interview received a monetary incentive in the equivalent of 14.2 USD. Interviews with physicians were conducted in their premises or NGO office. Interviews with social workers and TP were conducted at a convenient time in private and comfortable separate room at the NGO premises. Interviews lasted between 26 and 155 min with a mean duration of 80 min.

### Research team

Interviews were conducted by three independent interviewers, cisgender women with extensive experience working with LGBT community and the necessary skills and training in conducting interviews. The first interviewer has a Master’s degree in health care management and biology, and conducted 18 interviews. The second interviewer from the LGBT organization is a sociologist and conducted 14 interviews. The third interviewer, a psychologist with extensive experience in LGBT research, also conducted 14 interviews.

All authors are health and KPs researchers with necessary skills and training in conducting qualitative research and data analysis. Transcripts were manually coded by the first (ON) and the third (OK) author (i.e., coders). ON is a MD, has a Master degree in health management and Master degree in science. OK has a Master of Arts in Sociology, and has also completed training courses on WHO, СDС, University of Washington, and Harvard T.H. Chan School of Public Health, including analyzing qualitative data and conducting research among KPs.

### Data analysis

Interviews were digitally recorded, verbatim transcripts were carefully read and analyzed using the thematic analysis technique [18]. Transcripts were reviewed by the coders to conduct initial coding. Coders reviewed the data separately and then met, discussed and compared the coding. Common ideas among the codes were grouped into categories and into broad themes. Codebook was developed and included themes, categories, quotes, and definitions. Monthly meetings with all authors were held to discuss the themes until reaching a consensus on the theme development. The quantitative descriptive analysis of sociodemographic data was performed in SPSS 20 (IBM SPSS Statistics for Windows, Version 20.0. Armonk, NY: IBM Corp).

Several procedures were taken to ensure validity of the results [19]. Particularly, representatives of TP community were consulted to develop study materials, hold training and interpret findings. The interdisciplinary research team (i.e., public health, psychology, sociology, LGBT research, medicine) was able to consider a variety of factors influencing the analysis and findings. The study team met regularly to discuss the process of data collection and findings. The authors used reflexivity practices to critically examine their own biases regarding the community-based HIV prevention services for TP. Consensus among all authors, as well as input of representatives of TP community, helped ensure the validity of the results.

## Results

### Study sample characteristics

Of 6 social workers, there were 3 women and 3 men with the mean age of 32 years and mean work experience of 7 years. Of 10 physicians, there were 9 women with the mean age of 42 years and mean work experience of 15 years. By current position, there were 3 family doctors, 2 endocrinologists, 2 urologists, 2 psychiatrists and 1 infectious disease specialist. 30 TP were represented by TW (n = 20) and TM (n = 10) with the mean age of 30 years. Two thirds (n = 20) were never married, the majority (n = 29) reported at least high school diploma, more than a half (n = 19) were working or/and studying. Half of the TP reported HIV prevention services history, the majority (n = 24) had a hormone therapy history, 10 respondents reported gender-affirming surgery, and 2 TP were living with HIV. Detailed characteristics of study participants are presented in Tables 1 and 2.

**Table 1:** Characteristics of physicians and social workers

Social workers	Total (n = 6)
Variable	**n / mean**
Age, years (min – 24, max – 36)	32 (± 4,1)
Gender	
man	3
woman	3
City	
Kyiv	3
Odesa	3
Position	
social worker	3
head of the project	1
psychologist	1
lawyer	1
Work experience (years) (min − 0, max − 14)	7 (± 5,7)
**Physicians**	**Total (n = 10)**
**Variable**	**n / mean**
Age, years (min – 30, max – 62)	42 (± 9,6)
Gender	
man	1
woman	9
City	
Kyiv	5
Odesa	5
Position	
family doctor	3
endocrinologist	2
urologist	2
psychiatrist	2
infectious disease specialist	1
Work experience (years) (min – 6, max – 30)	15 (± 6,9)

**Table 2:** Characteristics of TP

Variable	TM (n = 10)		TW (n = 20)		Total (n = 30)	
	**n / mean**	**% / SD**	**n / mean**	**% / SD**	**n / mean**	**% / SD**
Age, years (min. – 18, max. – 48)	26	± 7,8	31	± 9,4	30	± 9,1
HIV prevention services history	5	50	10	50	15	50
Hormone therapy history	10	100	14	70	24	80
Gender-affirming surgery	5	50	5	25	10	33.3
Marital status						
never married	6	60	14	70	20	66.7
married	3	30	4	20	7	23.3
divorced	1	10	2	10	3	10
Education						
less than high school	0	0	1	5	1	3.3
high school diploma	4	40	8	40	12	40
some college or university	3	30	4	20	7	23.3
college or university degree	3	39	7	35	10	33.3
Work						
full time	2	20	7	35	9	30
part time	3	30	3	15	6	20
studying	1	10	1	5	2	6.7
studying + part time work	1	10	1	5	2	6.7
seeking for work	0	0	2	10	2	6.7
no work + do not seeking work	1	10	3	15	4	13.3
other	2	20	3	15	5	16.7
Positive HIV status	0	0	3	15	3	10

Legend of table: SD – standard deviation; Min. – Minimum; Max. – Maximum

### Thematic findings

#### Relevance of the community-based HIV prevention services to the needs of TP

A centralizing theory that emerged during the analysis was ‘*The needs related to gender transition’*. Four major themes emerged within the scope of the first research question.

#### Experience of TP on HIV prevention services utilization

Half of the respondents among TP were clients of HIV-prevention services. Most of them reported a positive experience in using these services based on the friendly attitude of social workers, as well as convenience while receiving the services. The existing package of services was mainly discussed in the context of receiving counseling and testing services, condoms and lubricants, and the ability to be referred to other services.

*‘…. she [author note – social worker] told me about what HIV is, how it can be transmitted, all the transmission routes … And from that moment, I try to get HIV test at least once every six months.’* (TW, 34 years).

### Sexual and injection risky behaviors of TP

All TP reported no experience of injecting drug use. Several TP reported irregular use of non-injectable drugs, mostly marijuana. According to three service providers, the practice of using non-injectable drugs, especially *‘hard drugs’* (informal aggregation term used by some participants for amphetamine. ecstasy, salts, cocaine, heroin and several other drugs except marijuana), is more prevalent for TP-commercial sex workers.

*‘Of those we know who use non-injecting drugs are trans women who are involved in sex work…’* (project leader, 33 years).

Several TP and service providers believe that TP have an increased risk of using heavy non-injecting drugs in the atmosphere of family and society social stress, as well as for easier communication with partners. Several TP have reported that the process of gender transition also may intensify internal tensions and lead to uncontrolled alcohol and drug use.

*‘I think the risk of getting hooked on some hard drugs is higher precisely because of this social pressure.’* (TW, 34 years).

Most TP characterized their own practices as safe and either had no sexual contacts at all for a long time or practiced monogamous relationships.

### Demanded components of HIV prevention services

HIV testing, condoms and lubricants distribution were defined as the most relevant service by social workers and TP with HIV prevention services history. Interest in HIV testing was connected with a perceived risk of HIV infection among several TP who practiced risky sexual behavior. TP consider condoms are valuable due to their cost.

*‘Well, other medical services are more important to me. Concerning HIV prevention, it is enough for me to be able to get tested [author note – HIV testing].’* (TM, 39 years).

For some TP HIV prevention services are not a priority, but the opportunity to receive health consultations and referrals to other services due to transgender transition. Many TP reported that they wanted to receive current HIV prevention services less often than it is offered, but if these services address their needs, they would receive them regularly.

*‘The services that I am telling now: endocrinologist, psychologist, lawyer, hormonal drugs support, laboratory examinations … This is more important for a transgender person than testing for HIV or condoms. Because we …, we strive for this, this is all costly…’* (TW, 29 years).

### TP needs related to gender transition

Many TP noted that they had to pay for gender transition related medical services, four TP reported payment for psychiatrist services, to be diagnosed. Applying for medical services, such as a sex hormone test, requires significant financial resources and can be performed in specialized health care facilities only.

*‘Regarding hormones, I visited her [author note - endocrinologist] two or three times, then I just ran out of money… (laughs).’* (TM, 20 years).

### Hormonal therapy

Many transgender participants, and several physicians, mentioned that one drug is used during hormonal therapy. All 10 TM reported hormone-affirming therapy history, mostly injectable testosterone.

*‘I teach them [author note – TP] how to inject, and not to have bumps, so that the drug does not fly out of the needle. They do not know that they need 40–45 degrees … water …, held the ampoule, quickly typed…’* (family physician, 47 years).

Many TP with hormone-affirming therapy history started taking hormone drugs on their own, without a doctor’s prescription. According to several TP and social workers, self-prescribing hormone therapy is easily accessible, cheaper and faster to receive. It is enough to get advice from other TP who had such an experience via internet forums and purchase *‘recommended’* drugs online.

*‘Recently, we had TP who generally makes these hormones at home, by buying various…, veterinary, including ingredients, mixing them… And she, not only takes them by herself, she still sends them to others transgender people…’* (project leader, 33 years).

Without regular monitoring by a physician, self-administration of hormone therapy often may not bring the expected result, which was discussed by many TP and several physicians. It pushes some TP to self-increase dosages and self-adjust drug regimens, relying only on subjective feelings and changes in appearance. Many TP and four physicians discussed that uncontrolled hormone therapy usage can cause side effects, which further deteriorate the health of TP.

*‘…psychological nervous conditions. …. varicose veins began to appear. And in general, there are many other such problems - shortness of breath, there was always some kind of fatigue. But again, this was all from the wrong hormones that I was taking, because I did not know, I used at random.’* (TW, 21 years).

The reasons why several TP stopped taking hormone therapy were health issues, the relations with family, stress factors and belief that the natural appearance is sufficient to be perceived as a woman.

*‘I would like to take hormone therapy and everything else … but I live in this society and I cannot explain it, for example, to my parents. Because they are elderly people. I do not want to hurt them.’* (TW, 39 years).

### Surgery

TP have different attitudes towards the need for transgender surgery: from a complete reluctance to an urgent need for some plastic or radical surgeries. The cost of such services plays a significant role in decision making which was discussed by many respondents. Mastectomy was reported as a way to improve the quality of life for TM and orchiectomy - to increase the effectiveness of hormone therapy for TW. If mastectomy is not possible, one of the alternative ways to change the appearance of TM as well as to reduce stress and anxiety is to wear so-called *‘binder’* to look like a person without breasts which was discussed by several TM. Special items or smaller size tops or bandages are used for *‘binder’*.

*‘It’s like a fabric thing that you put on like a T-shirt, I guess. And it tightly wraps around the chest to create the appearance of a flat chest. … And it, again, helps to reduce levels of depression, anxiety and, in general, stress in life.’* (TM, 19 years).

### Gender-responsive documents

This stage provides opportunities to sue the TPs right to social integration, employment, and full access to medical and social services. Insufficient information available on the gender transition algorithm was noted by many respondents. There are legal barriers to issuance of proper documents - a passport with an appropriate gender can be obtained only at the place of registration.

*‘They [author note - TP] do not know this algorithm, roughly speaking, where to start? The only problem so far is with a passport … it can be changed only at the place of registration.’* (lawyer, 32 years).

### Social component

Many TP reported that social component of the gender transition concerns the formation and consolidation of certain relations between own gender perception and social context, including by having a new name and personal pronouns that correspond to gender.

*‘When I changed my name, when I asked friends to call me in a certain way, when I told my parents about it…’* (TM, 19 years).

Based on most respondents, stigmatization and discrimination are important factors that can significantly complicate the gender transition process. Some physicians and many TP discussed the inadequate reaction and ridicule from other physicians to those who provide medical services to TP. Several TP reported violence from their surroundings as well as society. Many TP need to travel to another city to visit a doctor. Often, TP seeks medical care when their health condition becomes critical or hormonal drugs do not cause the desired result.

### The key components of the preferred “ideal” HIV-prevention package

According to the majority of respondents, a separate service package for TP should be developed, which will integrate gender transition, HIV prevention and other services. The main characteristics of this integrated package are shown in Table 3.

*‘If there was a psychologist, an endocrinologist or something else… Otherwise, why would I come here often? Well, I can come here once in 4 months to get tested.*’ (TW, 29 years).

**Table 3:** Characteristics of optimal HIV prevention package for TP

Component	Content description	Provision description
All components	Provision of services based on the needs of TP.	• Needs/risk assessment before service provision.
Counselling	• Counselling on transition. • Psychological counseling. • HIV prevention counseling for TP at high risk of HIV infection. • Consultation by physician at NGO office.	• Counseling using social networks and mobile applications, online counseling, hotline and counseling directly in the NGO office. • Feedback provision during the remote counseling, linkage to services. • Peer-to-peer counseling, online and face-to-face. • Provision of counselling by TP with positive experience of transition.
Referral and navigation	• Appointment with a doctor (if needed). • Referral and navigation (if needed) to medical services (prescription and monitoring of hormone replacement therapy, medical examination, enrolment in HIV care, etc.) and legal services (paperwork, etc.).	• Creation of the pool of friendly doctors who are ready to help TP, and therefore willing to receive the appropriate training. • Training of doctors on service provision for TP. • Training of multidisciplinary teams for provision of package medical services for TP.
Distribution of prevention tools and educational materials	• Distribution of information and educational materials on transition. • Provision of prevention tools that meet the needs of TP: lubricants in tubes, femidomas, latex wipes.	• Development of information materials related to transition, as well as materials on HIV and STIs prevention following the characteristics of sexual behaviours of TP. • Dissemination of printed information materials in NGO office and e-materials through the social networks.
HIV/STI testing	• HIV/ STI self-testing. • Partners’ testing (if needed and possible).	• Use of test kits that examine oral fluid.
PrEP	• PrEP in NGO office.	• Access to PrEP for TP at risk (sex workers, HIV-negative partners in discordant couples etc.) or with latex allergy. • Consultation on possible drug interactions (PrEP and hormone replacement therapy).
PEP	• Health care facility based PEP.	• Provision of information on the availability of PEP, referral and navigation to PEP for those at risk. • Consultation on possible drug interactions between (PEP and hormone replacement therapy).
Financial support	• Financial compensation for services related to transition.	Options for financial compensation: • Hormone testing. • Hormone replacement therapy. • Medical consultations. • Transition-related surgeries. • Document changing fees. • Hostel payment. • *‘Tightenings’* or other items for everyday living.
TB screening	• TB screening questionnaire.	If needed.

Legend of table: NGO – non-governmental organizations; PEP – post-exposure prophylaxis; PrEP – pre-exposure prophylaxis; STIs – sexually transmitted infections; TB – Tuberculosis; TP – transgender people.

Many TP need to be able to obtain information on the combination of preventive and medical services, such as pre-exposure prevention (PrEP) and gender-affirming hormone therapy (PrEP is not currently provided to TP in Ukraine). Some TP do not see the feasibility of using PrEP because it does not replace condom use. Based on physicians and several TP, there is a particular need in information about the interaction of hormonal drugs and ART.

‘When ART is prescribed to a person taking male hormones, there is no considerable interaction with our ARV drugs. But when a person takes female, that is, feminizing substitution therapy, in this case, surely, it’s not recommended to prescribe the drugs that have any impact on female hormones level…’

(physician, 43 years).

Some TP reported the urgency of obtaining legal advice in connection with the transgender transition, including changes to documents.

*‘Legal advice. On certain issues of interest to me, like changing documents, for example…’* (TW, 24 years).

### The ways to optimize existing HIV prevention package for TP

Based on many respondents’ social media, mobile applications, *“word-of-mouth”* approach with the involvement of *“opinion leaders of the community”* and TP as service providers may increase the involvement of TP in prevention services. The Internet provides an opportunity to get answers to most questions without leaving comfort zone for many TP. Online communication provides information and stories from *“peers”*. The so-called *“opinion leaders of the community”* play an important role in this regard - people who have something to say, know how to convey it and can unite others around themselves.

*‘… if, for example, I have some long-term cough or cold, I would seek an acquaintance who can listen to my lungs…Because I understand that when I go to a doctor and roll up my T-shirt, there will be questions…’* (TM, 39 years).

Attracting and retaining TP in HIV services will be more effective if prevention services can address their needs, or at least provide a link to gender transition-related services which was discussed by many respondents.

Service providers and TP noted the following ways for the existing HIV prevention programs optimization: psychological counseling or referral to a psychologist; referral and navigation to medical and legal services related to transition, or other medical services if needed; referral to PrEP and post-exposure prevention (PEP) if needed; dissemination of preventive means that meet the needs of TP (tubed lubricants, femidoms and latex wipes); use of oral fluid test systems for HIV self-testing.

*‘… to have support in visiting not only some AIDS center, but to other doctors as well.’* (TM, 39 years).

## Discussion

Most TP with HIV prevention services history expressed positive attitude towards receiving HIV prevention services. Several studies reported that many TP used these services as a gateway to other services including medical care [14]. Many TP in our study perceived the low risk of HIV acquisition and described specific needs in selected prevention components (HIV testing, condoms and lubricants). Based on IBBS results conducted in Ukraine among TP, HIV prevalence among TW with sex work experience was 6% versus 1% among TW without such experience [12].

The majority of respondents in our study described the low prevalence of injection drug use among TP. The results of IBBS among TP demonstrate that the vast majority of TW (86%) and TM (78%) reported alcohol consumption, 66% of respondents reported cannabis consumption [12].

The findings of our study show that many TP perceive the importance of gender transition services over the needs of HIV prevention services. The results of other studies also confirmed that gender affirmation was perceived by TP as the key health issue [20, 21]. It was reported that TW living with HIV may prioritize gender transition-related care over HIV-related care, especially early in their gender transition process [22]. In our study body modification using hormones or/and surgery was mostly perceived as the main components of medical gender transition. Based on the results from other studies, these therapies allow TP physical presentation meets their internally experienced gender identity [23, 24].

Our study reveals the high prevalence of self-administration of gender-affirming hormones and associated adverse reactions. High prevalence of hormone use without physician prescription is well-documented in other studies [24–28]. The results of the systematic review demonstrated that self-administration of gender-affirming hormone therapy may be due to lack of knowledgeable and non-stigmatizing health workers, lack of access to appropriate services, cost, and desire for a faster transition [27], which is relevant to our findings.

Our study results demonstrated that multiple interrelated barriers including stigma, discrimination, lack of providers’ competence, limited navigation, financial issues lead to self-prescribed hormone use, avoidance or postponing of medical care use. Other studies confirmed the role of stigma and discrimination, inadequate cultural competence of providers, lack of provider training on transgender healthcare as barriers to care provision [20, 24, 29, 30]. Limited access to gender-affirming surgery and its high cost was reported by the study participants, which was also described in other studies particularly in low-income countries [9, 14].

The *“ideal”* prevention package for TP may serve as a benchmark for planning and implementing prevention and care services for TP. The formulation of the targeted prevention package for TP and integration of gender transition, gender-affirming healthcare and HIV prevention, other services were discussed in this and other studies [31–34]. Our conclusions and other studies results showed that such integration may also include financial aid, job referral, legal services, psychological support and counseling [14]. It was reported in several studies that integration of HIV prevention, care services and hormone therapy for TP was highly acceptable and facilitated the engagement and uptake of HIV prevention services [22, 24, 35, 36].

Physicians in our study described PrEP as a promising intervention, however, the perception of TP in this regard was not unanimous. Other studies demonstrated the possibility of PrEP utilization among TP as a routine HIV protection regardless of partnership context and condom use [37, 38].

In other study social media, mobile applications and word-of-mouth approach were described as a way to improve enrollment in HIV prevention services as TP networked with other TP, using support groups and the Internet [30]. The study results among TW demonstrated that the majority of information regarding their health, hormones, risk reduction, and HIV prevention were received from each other [24]. The important role of TP leaders in the recruitment to services was reported as well [35].

Navigation to medical services was described as an important intervention component in our study. Other studies show that navigation can facilitate access and uptake of HIV and gender-affirmative services for TP [24].

Proper and consistent use of condoms is recommended by the World Health Organization (WHO) for the prevention of HIV and STI among all KPs [39]. The existing package of preventive services for TP in Ukraine meets this recommendation. Taking into account the regional context, the WHO recommends taking measures to reduce the possible harm of injectable hormone replacement therapy. Respondents of our study did not report the needs in injecting equipment. WHO also recommends targeted Internet interventions, social marketing strategies, and sex venue-based outreach. According to the respondents, the involvement of *“opinion leaders”* and provision of peer services may play an important role.

Finally, introduction of International Classification of Diseases and Related Health Problems-11 in Ukraine and removing gender identity disorder from the chapter will address several limitations in services provision. It will reduce TP’s fear of being automatically labelled mentally ill [9].

### Limitations

Most respondents among TP described their sexual behavior as safe either because of celibacy or safe sex practices. TP with the highest HIV infection risk may be underrepresented in this study. Underrepresentation of TP who are living with HIV does not allow to make conclusions regarding HIV care for TP. Our study was conducted in two cities with high HIV burden and high coverage of HIV prevention among TP, limiting its nationwide generalizability.

## Conclusions

This qualitative study provides a novel information on the current state, existing limitations and ways for improving the community-based HIV prevention services for TP in Ukraine. HIV prevention programs delivery, including engagement and retention TP, may be optimized by implementing targeted services that are TP oriented and integrate transition-related healthcare needs.

## Data Availability

The datasets used and/or analyzed during the current study are available from the corresponding author on reasonable request.

## List of Abbreviations

IBBS: Biobehavioral study
KPs: Key populations
NGOs: Non-governmental organizations
PEP: Post-exposure prophylaxis
PrEP: Pre-exposure prophylaxis
TP: Transgender people
TM: Transgender man
TW: Transgender woman
WHO: World Health Organization
